# Achaete scute-like 2 suppresses CDX2 expression and inhibits intestinal neoplastic epithelial cell differentiation

**DOI:** 10.18632/oncotarget.5206

**Published:** 2015-08-17

**Authors:** Yangyang Shang, Qiong Pan, Lei Chen, Jun Ye, Xiaoli Zhong, Xiaohuan Li, Linkuan Meng, Jin Guo, Yin Tian, Yonghong He, Wensheng Chen, Zhihong Peng, Rongquan Wang

**Affiliations:** ^1^ Department of Gastroenterology, Southwest Hospital, Third Military Medical University, Chongqing, P. R. China

**Keywords:** Achaete scute-like 2, CDX2, transcriptional regulation, colorectal carcinoma, differentiation

## Abstract

The role of Achaete scute-like 2 (Ascl2) in colorectal cancer (CRC) cell differentiation is unknown. LS174T, HT-29 and Caco-2 cells have high Ascl2 expression, while Lovo and SW480 cells have low Ascl2 expression. LS174T and HT-29 cells with Ascl2 knockdown were transfected with caudal type homeobox 2 (CDX2) promoter constructs and used for luciferase assays and chromatin immunoprecipitation (ChIP) assays. Ascl2 knockdown promoted differentiation of CRC cells into a goblet cell phenotype, as determined by increased expression of MUC2, TFF3, and CDX2. Ascl2 knockdown activated CDX2 expression through a transcriptional mechanism via direct binding of Ascl2 to the proximal E-box of the CDX2 promoter. Ascl2 over-expression in Lovo and SW480 cells inhibited a goblet cell phenotype, as determined by reduced CDX2 and MUC2 expression. Inverse correlations between Ascl2 and CDX2, and Ascl2 and MUC2 mRNA levels, as well as Ascl2 and CDX2 protein levels were observed in CRC cancerous samples. This study demonstrates CDX2 repression by Ascl2 and highlights a role for Ascl2 in CRC cell differentiation. These findings suggest that the Ascl2/CDX2 axis may serve as a potential therapeutic target in colorectal cancer.

## INTRODUCTION

A number of genes and encoded proteins participate in the maintenance of stemness and the differentiation of colorectal cancer (CRC) cells [1-3]. Understanding the regulatory mechanisms and signaling pathways involved in CRC stem cell differentiation is important for the development of novel drugs that promote this differentiation, while inhibiting stemness [3-5]. However, the molecular mechanisms bridging CRC stem cell maintenance and the induction of differentiation in CRC cells are largely unknown.

Achaete scute-like 2 (Ascl2), a basic helix-loop-helix (bHLH) transcription factor and downstream target of Wnt signaling, controls intestinal crypt stem cell fate [6, 7]. Ascl2 is over-expressed in colorectal cancer [6, 8, 9], shifting the hierarchy of stem and progenitor cells in liver metastases and results in self-renewal rather than differentiation [9]. Blockade of Ascl2 expression in HT-29 and LS174T cells results in tumor growth arrest via miRNA-302b-mediated inhibition of CRC progenitor cells and induces miR-200 family expression, further promoting epithelial-mesenchymal transition (EMT)-mesenchymal-epithelial transition (MET) plasticity via a transcriptional mechanism [10, 11]. Thus, Ascl2 may be a regulatory factor in the maintenance of CRC stem cell.

Caudal type homeobox 2 (CDX2) encodes an intestinal transcriptional factor of the homeoprotein family that is essential for the development and maintenance of the intestinal mucosal epithelium [12, 13]. CDX2 inhibits cell growth and stimulates cell differentiation in intestinal mucosal epithelial cells and CRC cells [14, 15]. CDX2 binds to the mucin 2 (MUC2) promoter, activating transcription, and stimulating the differentiation of goblet cells [16]. In this report, we demonstrate that the Ascl2/CDX2 axis promotes plasticity between stemness maintenance and differentiation in CRC cells and could be a potential target for the development of novel therapies.

## RESULTS

### Ascl2 deficiency in CRC cells promotes differentiation into a goblet cell phenotype

**Figure 1:**
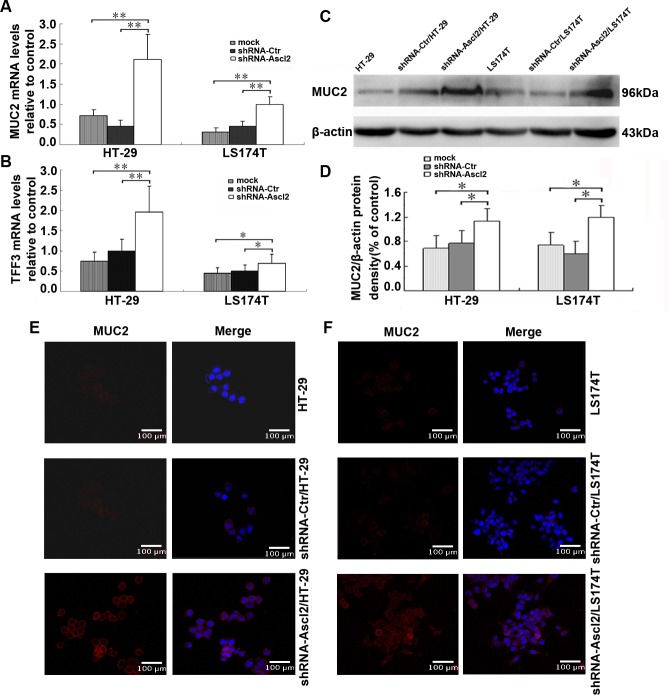
Ascl2 knockdown in CRC cell lines increased expression of goblet cell specific genes **A.** MUC2 mRNA levels in CRC cell lines (HT-29, shRNA-Ctr/HT-29, shRNA-Ascl2/HT-29, LS174T, shRNA-Ctr/LS174T and shRNA-Ascl2/LS174T cells) were quantitated by real-time PCR analysis twice, each in triplicate (*n* = 6). **B.** *TFF3* mRNA levels in CRC cell lines were quantitated by real-time PCR analysis twice, each in triplicate (*n* = 6). **C.** and **D.** MUC2 protein expression in CRC cell lines and further densitometric analysis (*n* = 3). **E.** and **F.** Immunofluorescence staining of MUC2 protein in CRC cell lines.

To determine whether Ascl2 deficiency in CRC cells can lead to their differentiation, we used qRT-PCR to quantify expression levels of cell-type specific genes in shRNA-Ascl2/HT-29 and shRNA-Ascl2/LS174T cells. Two genes specific to goblet cells, MUC2 and trefoil factor 3 (TFF3), were more highly expressed in shRNA-Ascl2/HT-29 and shRNA-Ascl2/LS174T cells when compared with their controls (Figure 1A-1B); however, two genes specific to paneth cells, phospholipase A_2_ group IIA (*PLA2G-2A*) and lysozyme, showed no alteration in expression (data not shown). Isomaltase and lactase (genes specific to the absorptive epithelium) and chomogranin A and Nero D1 (genes specific to enteroendocrine cells) were undetectable in either shRNA treated or control cells. Because the MUC2 protein is commonly used as a differentiation marker of goblet cells, we examined expression of the MUC2 protein using a polyclonal antibody that recognizes its carboxyl terminal domain. Western blot analysis of Ascl2-deficient colon cells showed increased MUC2 expression as compared to their control cells (Figure 1C-1D). Immunofluorescence staining of the MUC2 protein revealed increased expression in both shRNA-Ascl2/HT-29 and shRNA-Ascl2/LS174T cells as compared with control cells (Figure 1E-1F). Thus, Ascl2 deficiency in CRC cells leads to increased expression of goblet cell-specific genes, and promotes differentiation into a goblet cell phenotype.

### Ascl2 deficiency induces CDX2 expression in intestinal neoplastic epithelial cells

**Figure 2:**
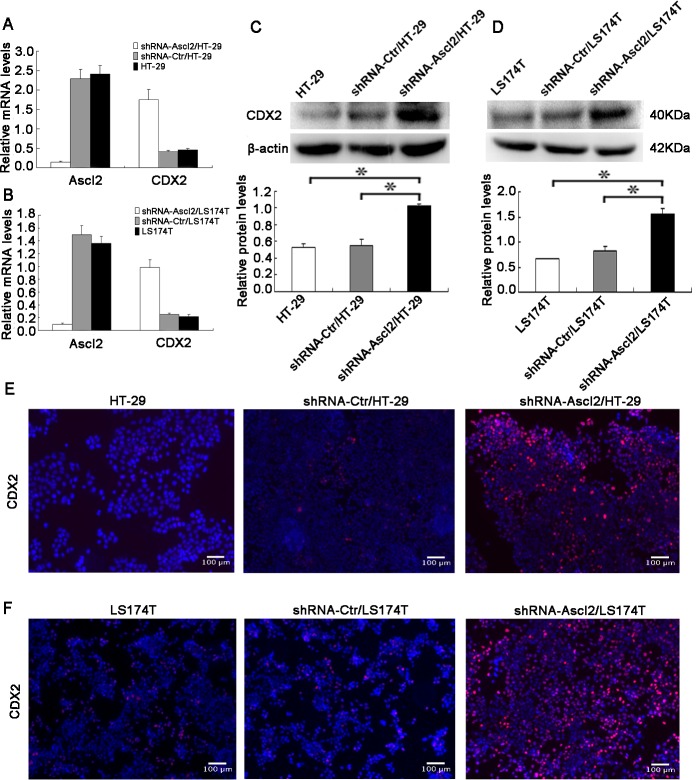
Ascl2 knockdown in CRC cell lines increased CDX2 expression **A.** and **B.** Inhibition of Ascl2 in HT-29 and LS174T cells led to significant increase in CDX2 mRNA levels which were quantitated by real-time PCR analysis twice, each in triplicate (*n* = 6). **C.** and **D.** Inhibition of Ascl2 in HT-29 and LS174T cells led to significant increases in CDX2 protein levels which were detected by Western blotting and further densitometric analysis (*n* = 3). **E.** Immunofluorescence staining showed stronger CDX2 expression in shRNA-Ascl2/HT-29 cells compared with controls. **F.** Immunofluorescence staining showed stronger CDX2 expression in shRNA-Ascl2/LS174T cells compared with controls.

CDX2 binds a *cis* element in the MUC2 gene promoter and activates transcription, and CDX2 over-expression stimulates the differentiation of goblet cells. Because Ascl2 deficiency in CRC cells led to their differentiation into a goblet cell phenotype and induced MUC2 expression, we hypothesized that it did so via increasing CDX2 expression. We used qRT-PCR to quantify expression of Ascl2 and CDX*2* in shRNA-Ascl2/HT-29 and shRNA-Ascl2/LS174T cells and their controls. When compared with control cells, Ascl2 expression was significantly decreased in shRNA-Ascl2/HT-29 and shRNA-Ascl2/LS174T cells, while CDX*2* expression was significantly increased (Figure 2A-2B). Similar increases in CDX2 protein levels in shRNA-Ascl2/HT-29 and shRNA-Ascl2/LS174T cells were observed in western blot analysis (Figure 2C-2D). Immunofluorescence staining of CDX2 in Ascl2-deficient CRC cells confirmed increased numbers of CDX2-positive cells, as well as increased CDX2 staining intensity in both shRNA-Ascl2/HT-29 and shRNA-Ascl2/LS174T cells when compared with control cells (Figure 2E-2F).

### Ascl2 represses CDX2 transcription

**Figure 3:**
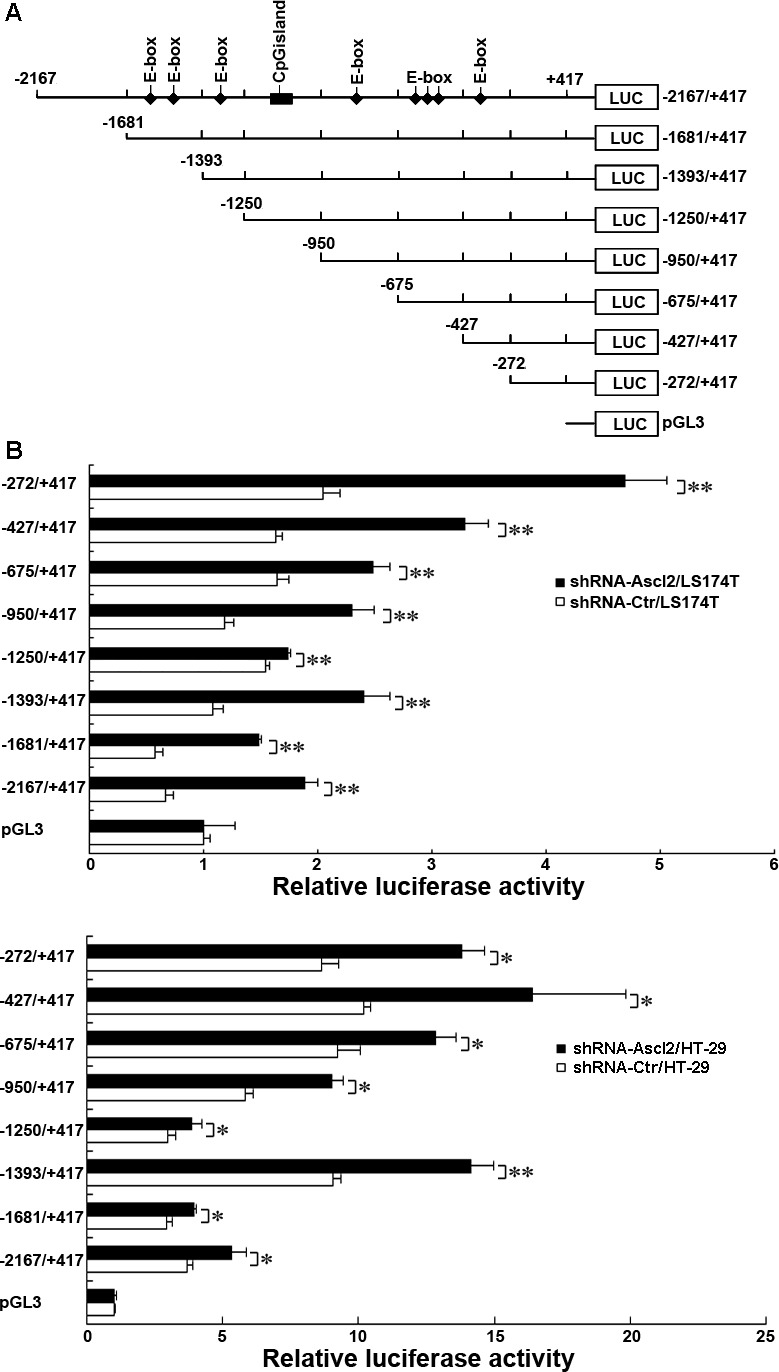
Transcriptional regulation of human CDX2 by Ascl2 **A.** A schematic representation of the human CDX2 promoter constructs used in this study. Seven promoter deletion-luciferase constructs were generated to identify the sites of transcriptional regulation within the human CDX2 promoter that respond to Ascl2 knockdown. **B.** shRNA-Ascl2/HT-29 and shRNA-Ctr/HT-29 cells were transfected with CDX2 constructs to identify the sites of transcriptional regulation within the human CDX2 promoter. **C.** shRNA-Ascl2/LS174T and shRNA-Ctr/LS174T cells were transfected with CDX2 constructs to identify the sites of transcriptional regulation within the human CDX2 promoter. Data are presented as the mean±S.E. of three independent experiments (*: *p* < 0.05; **: *p* < 0.01).

Ascl1, a homolog of Ascl2, was previously reported to form hetero-oligomers with the E12 transcription factor and can bind to the E-box of both muscle creatine kinase (MCK) and miRNA-200 *in vitro* [10]. Using promoter analysis, we found seven clustered E-boxes within the proximal 2167 bp region upstream of the transcription start site (TSS) of the human CDX2 gene (Figure 3A). To locate the specific regulators of CDX2 expression, the upstream amplifier region of CDX2 (−2167/+417) was inserted into a luciferase reporter pGL3 vector and truncated using relative primer pairs (Table 1): −1681/+417, −1393/+417, −1250/+417, −950/+417, −675/+417, −427/+417 and −272/+417 (Figure 3A). The full-length human CDX2 promoter (−2167/+417) generated a significantly higher level of luciferase activity in both shRNA-Ascl2/LS174T and shRNA-Ascl2/HT-29 cells than in control cells (*p* < 0.05 or *p* < 0.01; Figure 3B-3C). Significantly higher luciferase activity was also observed in shRNA-Ascl2/LS174T cells when using the pGL3-CDX2 promoter encompassing −1681/+417, −1393/+417, −1250/+417, −950/+417, −675/+41, and −427/+417 relative to the putative TSS (*p* < 0.01), in which different numbers of E-boxes were present (Figure 3C). Interestingly, the pGL3-CDX2 promoter encompassing −272/+417 relative to the putative TSS also had increased luciferase activity (*p* < 0.01) even with no potential E-box present. Identical experiments performed in shRNA-Ascl2/HT-29 cells and controls produced similar results (Figure 3B). These findings suggest that Ascl2 is a transcriptional repressor of CDX2.

**Table 1:** The primer sequences used in the each CDX2 promoter fragment

pGL3-CDX2-promoter	Primer pairs	Length of product
−2167/+417	F: 5′-TCTATCGATA GGTACC AGAGCCACGTCTTCAGG-3′ R: 5′-CTTAGATCGC AGATCT GCACGGAGCTAGGGTAC-3′	2584 bp
−1681/+417	F: 5′-TCTATCGATA GGTACC GGTCTCCCACCTCTTG-3′ R: 5′-CTTAGATCGC AGATCT GCACGGAGCTAGGGTAC-3′	2098 bp
−1393/+417	F: 5′-TCTATCGATA GGTACC GATGAAGGCGATGGTGAC-3′ R: 5′-CTTAGATCGC AGATCT GCACGGAGCTAGGGTAC-3′	1810 bp
−1250/+417	F: 5′-TCTATCGATA GGTACC TCGCCGCAGTGATCCT-3′ R: 5′-CTTAGATCGC AGATCT GCACGGAGCTAGGGTAC-3′	1667 bp
−950/+417	F: 5′-TCTATCGATA GGTACCAGTTGCCTTATCATCTCCT-3′ R: 5′-CTTAGATCGC AGATCT GCACGGAGCTAGGGTAC-3′	1367 bp
−675/+417	F: 5′-TCTATCGATA GGTACC TAACCACTAGCCTAACTTCT-3′ R: 5′-CTTAGATCGC AGATCT GCACGGAGCTAGGGTAC-3′	1092 bp
−427/+417	F: 5′-TCTATCGATA GGTACC ACAGGGCGGGAAGGAAT-3′ R: 5′-CTTAGATCGC AGATCT GCACGGAGCTAGGGTAC-3′	844 bp
−272/+417	F: 5′-TCTATCGATA GGTACC CAACCACTGCTCCTGTCT-3′ R: 5′-CTTAGATCGC AGATCT GCACGGAGCTAGGGTAC-3′	689 bp

The underlined parts in the primers are KpnI and BglII adaptors.

### Ascl2 binds the CDX2 promoter

**Figure 4:**
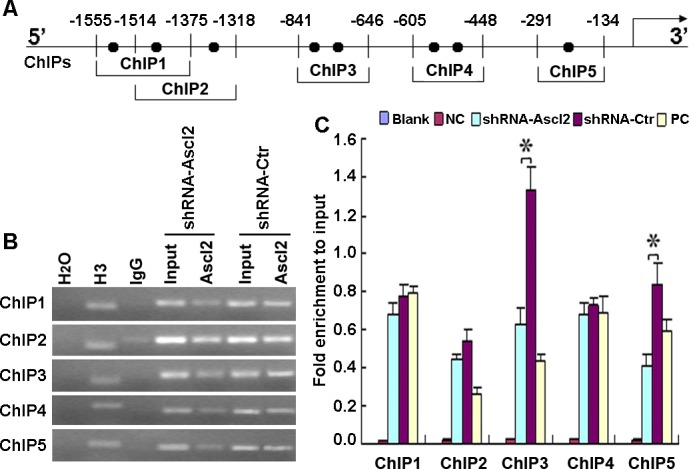
Ascl2 binding to the human CDX2 promoter Chromatin isolated from shRNA-Ascl2/LS174T and shRNA-Ctr/LS174T cells was subjected to immunoprecipitation using H_2_O (negative control), IgG antibody (negative control), anti-histone H3 antibody (mAb) (positive control) and mouse monoclonal IgG against Ascl2 (Millipore, MAB4418). The input represents 10% of the DNA used in the immunoprecipitation. **A.** Five sites in the CDX2 promoter with different numbers of E-box elements (ChIPs 1-5) were tested. **B.** The final DNA extracts were PCR-amplified using primers. **C.** The enrichment of the indicated genomic DNA fragments (ChIPs 1-5), the intergenic control (PC) or unspecific binding (blank and NC) was determined relative to the diluted input in three independent experiments.

We performed chromatin immunoprecipitation assay (ChIP) assays to determine whether Ascl2 binds directly to the CDX2 promoter and whether this binding decreased with Ascl2 knockdown in LS174T cells. As shown in Figure 4A, different numbers of E-boxes are present in the proximal promoters of CDX2. ChIP experiments 1-5 provided evidence of Ascl2 binding to these proximal promoters based on (Figure 4B). ChIP experiments 3 and 5 indicated that Ascl2 binding to the CDX2 promoter was reduced in shRNA-Ascl2/LS174T cells compared to that in shRNA-Ctr/LS174T cells at promoter positions −841/−646 and −291/−134. (Figure 4C). These results provide evidence of Ascl2 binding to the proximal promoter of CDX2.

### The CDX2 promoter has an Ascl2 cis-binding element

**Figure 5:**
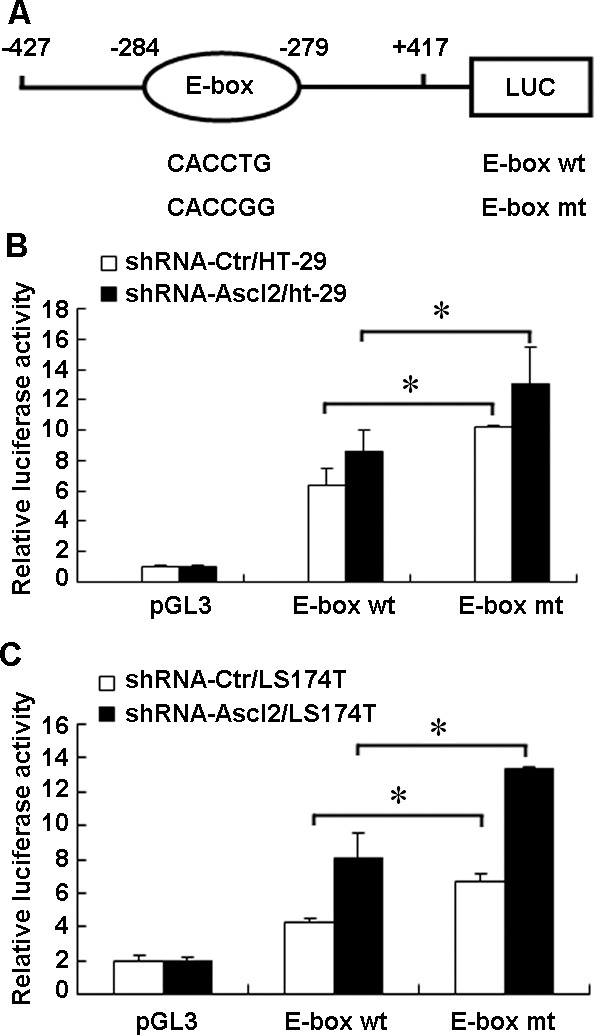
Identification of the cis-element that Ascl2 binds in the CDX2 promoter **A.** A schematic representation of the CDX2 promoter-Luc construct (−427/+417), which contains one E-box (CACCTG), and its mutant (CACCGG). **B.** shRNA-Ascl2/HT-29 and shRNA-Ctr/HT-29 cells were transfected with the CDX2 promoter-Luc construct (−427/+417) and its mutant to identify the sites of transcriptional regulation by Ascl2. **C.** shRNA-Ascl2/LS174T and shRNA-Ctr/LS174T cells were transfected with the CDX2 promoter-Luc construct (−427/+417) and its mutant to identify the sites of transcriptional regulation by Ascl2. The relative luciferase activity was determined using a dual-luciferase reporter assay system with a single sample luminometer. The data represented the mean±S.E. of three independent experiments (*: *p* < 0.05).

To determine whether Ascl2 transcriptionally suppressed CDX2 expression via binding to the E-boxes in the proximal promoter, we used the CDX2 promoter-Luc construct (−427/+417), in which only one E-box is present, to produce an E-box mutant (Figure 5A). shRNA-Ascl2/HT-29 cells or shRNA-Ctr/HT-29 cells were transfected with either the wild-type CDX2 promoter-Luc construct (−427/+417) or with the mutant construct. Both the wild-type and mutant CDX2 promoter-Luc constructs (−427/+417) showed significantly higher level of luciferase activity in shRNA-Ascl2/HT-29 cells compared to shRNA-Ctr/HT-29 cells (*p* < 0.05; Figure 5B). Similar results were found in shRNA-Ascl2/LS174T cells compared to shRNA-Ctr/LS174T cells (*p* < 0.05; Figure 5C). These results indicate that the most proximal E-box in the promoter of CDX2 functions as one of the binding sites for Ascl2 and that Ascl2 binding transcriptionally represses CDX2 expression. However, possible Ascl2 binding sites residing −272/+417 relative to the putative TSS of CDX2, which, when bound with Ascl2 still functioned as transcriptional repressors, remain unidentified.

### Ascl2 over-expression in colon cancer cells suppresses CDX2 and MUC2

**Figure 6:**
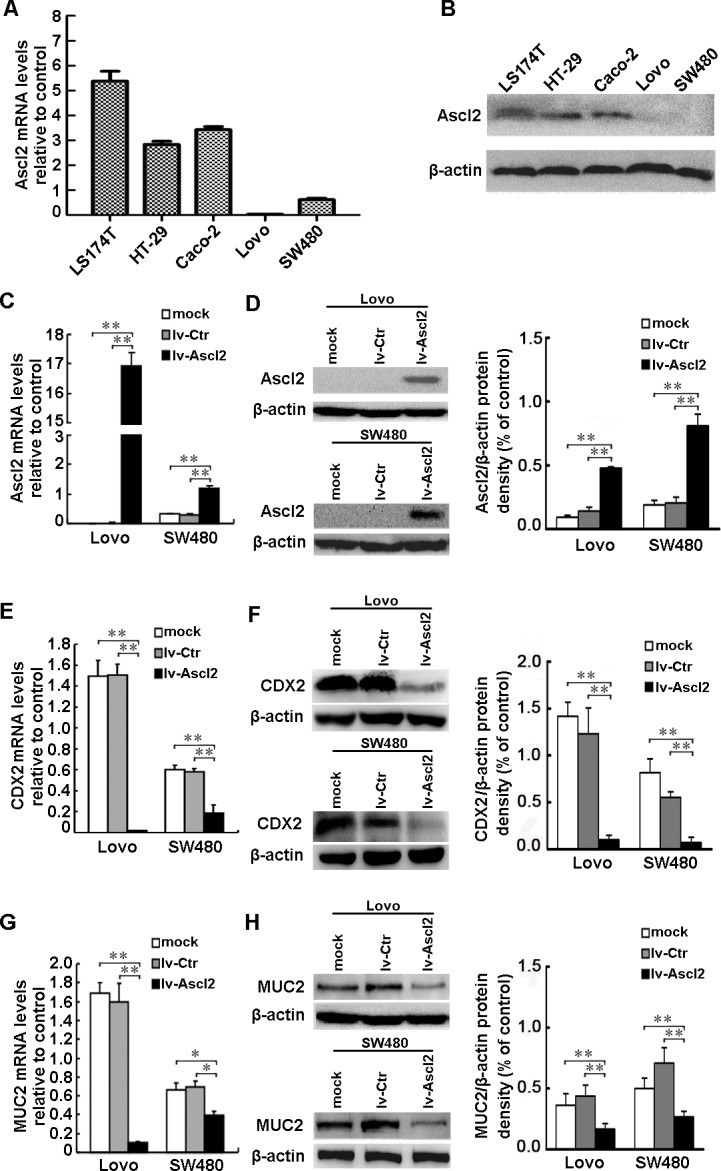
Ascl2 over-expression in CRC cells suppressed CDX2 and MUC2 expression Ascl2 mRNA and protein levels in HT-29, LS174T, Caco-2, Lovo and SW480 cell lines were detected via real-time PCR analysis and Western blotting assay. **A.** and **B.** Ascl2 mRNA and protein expression levels were lower in both Lovo and SW480 cells compared with their higher expression in HT-29, LS174T and Caco-2 cells. **C.** and **D.** Ascl2 mRNA and protein expression levels in lv-Ascl2/Lovo and lv-Ascl2/SW480 cells were significantly increased compared with lv-Ctr/Lovo and lv-Ctr/SW480 cells (*n* = 3). **E.** and **F.** CDX2 and MUC2 mRNA and protein expression levels were significantly reduced in lv-Ascl2/Lovo and lv-Ascl2/SW480 cells when compared with their negative control cells (*n* = 3). **G.** and **H.** MUC2 mRNA and protein expression levels were significantly reduced in lv-Ascl2/Lovo and lv-Ascl2/SW480 cells when compared with their negative control cells (*n* = 3). The data represented the mean±S.E. of three independent experiments (*: *p* < 0.05; **: *p* < 0.01).

Ascl2 mRNA and protein levels were measured in HT-29, LS174T, Caco-2, Lovo, and SW480 human colonic adenocarcinoma cell lines. Ascl2 mRNA and protein were lower in both Lovo and SW480 cells compared with HT-29, LS174T, and Caco-2 cells (Figure 6A and 6B). Thus, Lovo and SW480 cells were transfected with lentivirus particles expressing Ascl2, and stably transfected cells selected with puromycin. This resulted in four lines: lv-Ascl2/Lovo, lv-Ascl2/SW480 cells, lv-Ctr/Lovo, and lv-Ctr/SW480 cells. lv-Ascl2/Lovo and lv-Ascl2/SW480 cells had increased Ascl2 mRNA and protein expression levels as compared to lv-Ctr/Lovo and lv-Ctr/SW480 cells (Figure 6C and 6D). CDX2 (Figure 6E and 6F) and MUC2 (Figure 6G and 6H) mRNA and protein expression levels were significantly reduced in lv-Ascl2/Lovo and lv-Ascl2/SW480 cells when compared with their negative control cells. These results provide further evidence that Ascl2 expression represses CDX2 and, in turn, MUC2 expression.

### Ascl2 mRNA levels are inversely correlated with the CDX2 and MUC2 mRNA levels in CRC samples

**Figure 7:**
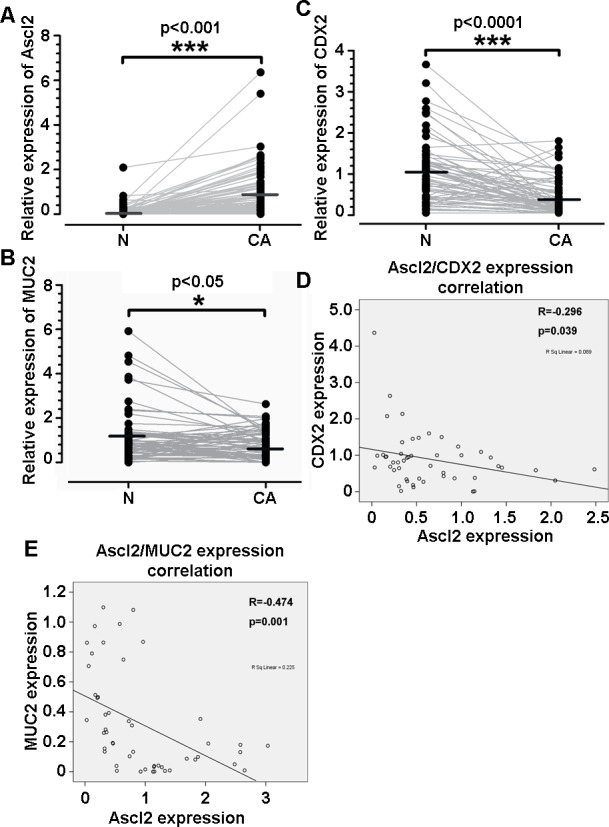
Correlations between Ascl2 mRNA levels and CDX2 and MUC2 mRNA levels in human colorectal carcinoma tissues Quantitative real-time PCR was performed in CRC tissues (CA) and their pericancerous mucosa (N) to assess Ascl2, CDX2, and MUC2 mRNA levels. Ascl2 expression was higher in CRC samples **A.**, whereas CDX2 **B.** and MUC2 **C.** expression was lower in CRC samples when compared with that in pericancerous mucosa. Ascl2 expression in CRC tissues was inversely correlated with CDX2 **D.** and MUC2 **E.** expression.

To verify whether Ascl2 can repress CDX2 gene expression in human colorectal cancer, quantitative real-time PCR was used to measure Ascl2, CDX2, and MUC2 mRNA levels in 50 CRC samples and their corresponding pericancerous mucosa. These samples were obtained from colorectal cancer patients who underwent biopsy via colonoscopy (*n* = 29) or surgical resection (*n* = 21). Ascl2 mRNA levels in the CRC samples were significantly higher than in the pericancerous mucosa (Figure 7A) (*p* < 0.001), whereas CDX2 (*p* < 0.05) and MUC2 (*p* = 0.0001) mRNA levels in the CRC samples were lower than those in the pericancerous mucosa (Figure 7B and 7C). Moreover, Ascl2 mRNA expression levels were inversely correlated with levels of CDX2 (*p* = 0.039) and MUC2 (*p* = 0.001) in CRC samples (Figure 7D and 7E). These results indicate that Ascl2, CDX2, and MUC2 are differentially expressed in human CRC tissues, and that their mRNA expression levels are inversely correlated.

### Ascl2 and CDX2 protein expression is inversely correlated in CRC samples

**Figure 8:**
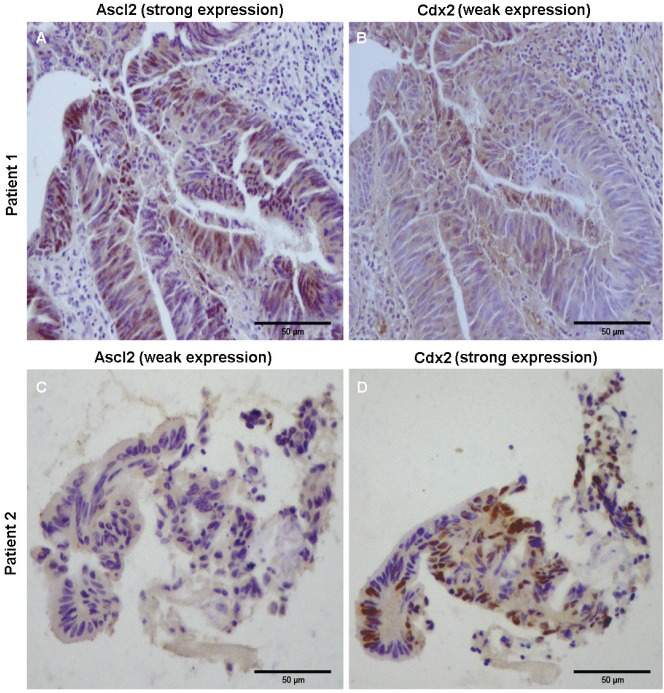
Immunohistochemical staining of Ascl2 and CDX2 proteins in cancerous tissues of colon cancer patients Ascl2 **A.** and **C.** and CDX2 **B.** and **D.** proteins were immunohistochemically stained in the cancerous tissues of two different colon cancer patients. Strong Ascl2 staining and weak CDX2 staining was found in the nuclei of cancerous cells of patient 1 **A.** and **B.**. In contrast, strong CDX2 staining and weak Ascl2 staining was found in the nuclei of cancerous cells of patient 2 **C.** and **D.**.

Immunohistochemical staining of Ascl2 and CDX2 proteins was performed in 21 cancerous samples from the above-mentioned patients. There was a significant relationship between Ascl2 and CDX2 protein expression in CRC samples: samples with high Ascl2 protein expression had low CDX2 protein expression, while samples with low Ascl2 protein expression had high CDX2 protein expression. As an example, Ascl2 protein was strongly expressed in the cancerous tissue from patient 1 (Figure 8A), while weak CDX2 protein expression was found in the same location from the continuous section (Figure 8B). In contrast, Ascl2 protein was weakly expressed in the cancerous tissue from patient 2 (Figure 8C), but extremely strong CDX2 protein expression was found in the same location from the continuous section (Figure 8D). These results suggest that Ascl2 and CDX2 expression in CRC tissues are inversely correlated at the protein level.

## DISCUSSION

In this study, we provide the first demonstration that Ascl2 acts as a putative transcriptional repressor of CDX2 in CRC cells, with direct implications for intestinal differentiation. Ascl2 is a downstream target of Wnt signaling in intestinal stem cells [7], and the Wnt signaling pathway is thought to be involved in intestinal cryptic Lgr5(+) stem cell fate [17, 18]. We reported previously that Ascl2 maintains stemness and controls the fate of CRC progenitor cells via miRNA-302b and miRNA-200 by maintaining plasticity of EMT and MET programs [10, 11]. Maintaining the stemness of CRC stem cells leads to inhibition of cellular differentiation; however, there has been a lack of molecular-level evidence of a direct association between these processes [3]. In the present study, HT-29 and LS174T cells were selected because these cells have high levels of endogenous CDX2, which is responsive to different molecular stimuli [19, 20]. Ascl2 is also abundantly expressed in these two cell lines, and was identified as regulator of CDX2 transcription. Ascl2 knockdown in CRC cells led to significant increases in the levels of goblet cell differentiation markers, including MUC2 and TFF3, and to marked increases in CDX2 mRNA and protein expression. The negative regulation of CDX2 by Ascl2 in CRC cells was confirmed using luciferase and ChIP assays which showed that Ascl2 binds to the CDX2 proximal promoter E-boxes. Ascl*2* over- expression in Lovo and SW480 cells lead to decreased expression of MUC2 and CDX2. Conversely, inhibition of Ascl2 expression in HT-29 and LS174T cell lines increased expression of two goblet cell-specific genes, MUC2 and TFF3, along with CDX2.

Ascl2 may have a dual role as a repressor or enhancer contingent on the cellular microenvironment. Endogenous Ascl2 expression is primarily localized in the nuclear compartment of intestinal Lgr5(+) cryptic stem cells and CRC cells. CDX2 protein level is lower in the crypts compared with that in the uppermost differentiated cells of the villi. The inverse distribution patterns of Ascl2 and CDX2 along the entire crypt-villus axis of intestinal mucosa, along with the inverse intensities of Ascl2 and CDX2 expression in CRC tissues indicates their inverse relationship [21, 22]. Our study indicates that Ascl2 not only fine-tunes CDX2 levels *in vitro* but also may regulate CDX2 *in vivo*. Moreover, intestinal Lgr5(+) cryptic stem cells are depleted in Ascl2 knockout mice [7], while Cdx2 knockout results in a reduction in the differentiated cells in intestinal mucosa [14, 23].

In human CRC, Ascl2 is predominantly expressed in CD133(+) progenitor cells [10], and CDX2 is decreased during the development of CRC [24, 25]. Furthermore, Ascl2 mRNA and protein levels are inversely correlated with the CDX2 levels in CRC samples. This indirect *in vivo* evidence, together with the *in vitro* results, strengthens our hypothesis for a role of Ascl2 in the control of intestinal cell differentiation via CDX2. Furthermore, the Ascl2-CDX2 axis may be important during CRC development because CDX2 is a recognized tumor suppressor gene of CRC [26, 27].

## MATERIALS AND METHODS

### Cell culture

The HT-29, LS174T, Caco-2, Lovo and SW480 human colonic adenocarcinoma cell lines were obtained from the Chinese Academy of Sciences Cell Bank of Type Culture Collection (Shanghai, China) and maintained at 37°C and 5% CO_2_ in McCoy's 5A medium (Sigma, USA) containing 10% fetal bovine serum (FBS) (HyClone, USA). The shRNA-Ctr/HT-29 cells, shRNA-Ascl2/HT-29 cells, shRNA-Ctr/LS174T cells and shRNA-Ascl2/LS174T cells were described previously and maintained in our lab [10].

### Tissue samples

Fifty patients with colorectal cancer who were scheduled for colonoscopy or for surgical resection were enrolled in this study. Cancerous samples and their pericancerous mucosa were collected by biopsy or from the resection samples. The fresh samples were immediately stored in liquid nitrogen for the further quantitation of Ascl2, CDX2 and MUC2 mRNA levels using quantitative real-time RT-PCR analysis. This study was approved by the local clinical research ethics committee. All of the subjects provided informed consent before their colonoscopy or resection surgery.

### Real-time PCR analysis

To determine the fold changes for each gene, real-time PCR was performed using first-strand cDNA, forward and reverse primers and the SYBR premix Ex Taq^TM^ Green II (TaKaRa, Japan). The primer sequences are summarized in Table 2. Reactions and signal detection were measured using a real-time PCR system (Bio-Rad, USA). Real-time PCR reactions were performed independently and in triplicate. Expression levels were calculated as the relative expression ratio compared to *ACTB* or *GAPDH* and relative mRNA expression levels calculated by the formula 2^−ΔΔCt^ using SDS software (Applied Biosystems).

**Table 2:** The primer sequences used in the real-time PCR experiment

	Primer pairs	Length of products
Ascl2	Forward: 5′-CGTGAAGCTGGTGAACTTGG-3′ Reverse: 5′-GGATGTACTCCACGGCTGAG-3′	113 bp
CDX2	Forward: 5′-CAGGACGAAAGACAAATATC-3′ Reverse: 5′-GATGTAGCGACTGTAGTG-3′	85 bp
MUC2	Forward: 5′-GAAGCCAGATCCCGAAACCA-3′ Reverse: 5′-GAATCGGTAGACATCGCCGT-3′	81 bp
TFF3	Forward: 5′-AGCCAAGGACAGGGTGGACT-3′ Reverse: 5′-GCTTGAAACACCAAGGCACTC-3′	110 bp
Lysozyme	Forward: 5′-GGGCTTGTCCTCCTTTCTGTTAC-3′ Reverse: 5′-CACATCCAGTTTGCTAGGCTGA-3′	122 bp
PLA2G-2A	Forward: 5′-ACGTCTGGAGAAACGTGGATGT-3′ Reverse: 5′-GCAGCCTTATCACACTCACACAG-3′	126 bp
Isomaltase	Forward: 5′-GCACTGTTATCCGACCCCTTT-3′ Reverse: 5′-GTAGTCAAACCACCGAGCATTG-3′	164 bp
Lactase	Forward: 5′-GTAGGAGGCTGGGAGAATGAGAC-3′ Reverse: 5′-CCCTGGTAAGCAATGACAAAGG-3′	131 bp
Chomogranin A	Forward: 5′-TGTCCTGGCTCTTCTGCTCTG-3′ Reverse: 5′-CTTGGAAAGTGTGTCGGAGATG-3′	124 bp
Nero D1	Forward: 5′-GACGAGTGTCTCAGTTCTCAGGA-3′ Reverse: 5′-TCTTCTTCCTCCTCTTCCAGGTC-3′	146 bp
β-actin	Forward: 5′-GTGATCTCCTTCTGCATCCTGT-3′ Reverse: 5′-CCACGAAACTACCTTCAACTCC-3′	132 bp

### Western blot assay

Cell lysates or homogenized tissues from tumor xenografts dissolved in SDS sample buffer were separated by SDS-PAGE and transferred to a nitrocellulose membrane. β-actin was used as a control. The membrane was probed overnight at 4°C with a specific primary antibody (rabbit monoclonal anti-CDX2 (ab76541), 1:200, Abcam; rabbit polyclonal anti-MUC2, 1:100, kindly provided by Dr. Forstner JF; monoclonal anti-Ascl2 (mab4418), 1:350). Detailed western blotting procedures were described previously (11).

### Transfection and luciferase assays

Fragments of the CDX2 5′-flanking sequence (−2167/+417 bp region) were amplified using PCR and cloned into the luciferase reporter vector pGL3-Basic (Promega, Madison, WI). Briefly, primers containing KpnI and BglII adapters were used to amplify the CDX2 promoter sequence from intestinal tissue DNA. The primer pairs used to produce each promoter fragment are listed in Table 2. The products were ligated into the pGL3-Basic vector that was digested with KpnI and BglII. The 5′ serial deletions of the −2584 bp CDX2 promoter region were generated using the Erase-a-Base system (Promega) in accordance with the manufacturer's recommendations. Plasmids for transient transfections were purified using an EndoFree Plasmid Maxi Kit (Qiagen, Valencia, CA). The day before the transfection, the cells were plated on 24-well plates at a density of 5×10^4^ cells per well. The CDX2 promoter-luciferase constructs were transfected into the cells using Lipofectamine^TM^ 2000 (Invitrogen). To normalize for transfection efficiency, cells were simultaneously co-transfected with a pRL-TK vector expressing the *Renilla* luciferase enzyme (pRL, Promega). The cells were harvested after 24 h, and the luciferase activity was measured using the Dual-Luciferase Reporter Assay System (Promega) and a single sample luminometer. The cells were also transfected with the pRL-TK vector; CDX2 activity is presented as the percentage of pGL3-control activity. The pGL3-Basic vector containing the CDX*2* promoter (−427/+417) and one E-Box site (CACCTG) served as a wild-type construct for the generation of the CDX2-Luc construct, which harbors a mutation in the E-BOX site (CACCGG) via PCR-based site-directed mutagenesis. Luciferase reporter transfection with different CDX2 promoters and luciferase assays were performed as described in our previous report (11).

### ChIP assay

ChIP assays were performed using a ChIP assay kit (Upstate Biotechnology, Lake Placid, NY) according to the manufacturer's instructions. Soluble chromatin was prepared from shRNA-Ascl2/LS174T cells or shRNA-Ctr/LS174T cells. Chromatin was immunoprecipitated with an antibody against Ascl2 (mouse monoclonal IgG, Millipore). The final DNA extracts were amplified by PCR using primer pairs that included different numbers of the E-Box consensus sequence in the human CDX2 promoter. The primer sequences and the lengths of the amplified PCR products are presented in Table 3.

**Table 3:** The primer sequences used in the ChIP experiment of CDX2 promoter

ChIP	Primer pairs	Length of products
ChIP1	Forward: 5′-GGCTAGTTCTGATCGCTTTC-3′ Reverse: 5′-TGTCACCATCGCCTTC-3′	181bp
ChIP2	Forward: 5′-GCTGGTTGTCACCTGTAAAA-3′ Reverse: 5′-CACTCCTGGAGACCTGC-3′	197bp
ChIP3	Forward: 5′-TACCTAGGTAAGCATTAGCA-3′ Reverse: 5′-GGCATGTGGTAGAAGTTAG-3′	196bp
ChIP4	Forward: 5′-AACGTTTAACAATAAATCGC-3′ Reverse: 5′-CAGCCCCAAACAACT-3′	158bp
ChIP5	Forward: 5′-TTCCCTGCAAATGCATAAAC-3′ Reverse: 5′-AGCTTCCTCCTTCCAACC-3′	158bp
ChIP6	Forward: 5′-TCGACGTCTCCAACCATT-3′ Reverse: 5′-CCTCCTTCCCACTAGGCT-3′	145bp
ChIP for positive control (GAPDH)	Forward: 5′-TACTAGCGGTTTTACGGGCG-3′ Reverse: 5′-TCGAACAGGAGGAGCAGAGAGCGA-3′	166bp

### Immunofluorescence cytochemistry and immunohistochemistry

Cells grown on coverslips were fixed in 4% paraformaldehyde for 30 min at room temperature and permeabilized with 0.5% Triton X-100. After blocking with 2.5% BSA in PBS (blocking solution) for 30 min, the cells were incubated with primary antibodies diluted in blocking solution overnight at 4°C (rabbit monoclonal anti-CDX2 (ab76541), 1:200, Abcam; rabbit polyclonal anti-MUC2, 1:100, kindly provided by Dr. Forstner JF). Then, the cells were washed three times with PBS and incubated with 50 μl Texas Red-conjugated (anti-mouse) or Alexa Fluor 647-conjugated (anti-rabbit) secondary antibodies (1:100 in blocking solution, Santa Cruz) at room temperature for 1 h in the dark. Monolayers were washed with PBS, and nuclei were stained with 50 μL DAPI (4′,6-diamidino-2-phenylindole; 1:2000 in PBS) solution for 2 min at room temperature. Then, tissue culture filters housing the epithelial cell monolayers were carefully detached from their support and mounted on coverslips. Immunostaining was analyzed using a Leica TCS SP5 confocal microscope. The CRC tissues were immunohistochemically stained with anti-Ascl2 or anti-CDX2 antibodies.

### Ascl2 over-expression assay

Lentivirus particles expressing Ascl2 were produced by GenePharma Co. Ltd (Shanghai, China). Lovo and SW480 cells were transfected with lentivirus particles using LV5 (EF-1aF/GFP&Puro) vector with Ascl2 insert. Stably transfected cells with GFP were sorted with a flow-cytometric sorting system (BD FACS Aria II; BD Biosciences, Franklin Lakes, NJ, USA) or isolated under puromycin selection (Solarbio, Beijing, China).

### Statistical analysis

For continuous variables, data are expressed as the mean±standard deviation. Differences between groups were estimated using Student's *t*-test and repeated-measures ANOVA analysis. All differences were deemed significant when *p* < 0.05. The correlations between Ascl2 mRNA levels and CDX2 or MUC2 mRNA levels in the cancerous samples were calculated by Spearman's rank correlation test. The statistical analyses were performed using the SPSS 13.0 for Windows software package.
